# Risk factors and a predictive model for under-five mortality in Nigeria: evidence from Nigeria demographic and health survey

**DOI:** 10.1186/1471-2393-12-10

**Published:** 2012-02-29

**Authors:** Gbenga A Kayode, Victor T Adekanmbi, Olalekan A Uthman

**Affiliations:** 1 Department of Public Health & Biostatistics, University of Birmingham, Birmingham, UK; 2 Centre for Evidence-Based Global Health, Ilorin, Kwara State, Nigeria

## Abstract

**Background:**

Under-5 mortality is a major public health challenge in developing countries. It is essential to identify determinants of under-five mortality (U5M) childhood mortality because these will assist in formulating appropriate health programmes and policies in order to meet the United Nations MDG goal. The objective of this study was to develop a predictive model and identify maternal, child, family and other risk factors associated U5M in Nigeria.

**Methods:**

Population-based cross-sectional study which explored 2008 demographic and health survey of Nigeria (NDHS) with multivariable logistic regression. Likelihood Ratio Test, Hosmer-Lemeshow Goodness-of-Fit and Variance Inflation Factor were used to check the fit of the model and the predictive power of the model was assessed with Receiver Operating Curve (ROC curve).

**Results:**

This study yielded an excellent predictive model which revealed that the likelihood of U5M among the children of mothers that had their first marriage at age 20-24 years and ≥ 25 years declined by 20% and 30% respectively compared to children of those that married before the age of 15 years. Also, the following factors reduced odds of U5M: health seeking behaviour, breastfeeding children for > 18 months, use of contraception, small family size, having one wife, low birth order, normal birth weight, child spacing, living in urban areas, and good sanitation.

**Conclusions:**

This study has revealed that maternal, child, family and other factors were important risk factors of U5M in Nigeria. This study has identified important risk factors that will assist in formulating policies that will improve child survival.

## Background

Childhood mortality is a prominent public health issue. Globally, mortality in under-five age children is about 9 million deaths per year and 70% are preventable which makes this an important public health problem to investigate [1]. In one of the series on child survival published on Lancet, childhood mortality was described as public-health disaster [2]. Children in developing countries are 10 times as more likely to die before their fifth birthday compared to those in developed countries [1]. The gap in childhood mortality between developed nations and developing nations has been increasing over a long period of time [3]. In response to the burden of childhood mortality in sub-Saharan Africa countries, United Nations and World Health Organization (WHO) has incorporated reduction of childhood mortality by two-third by the year 2015 as one of its Millennium Development Goals (MDGs).

Identifying determinants of under-five mortality (U5M) is essential for formulating appropriate health programmes and policies in order to meet the United Nations MDG goal (i.e. to reduce childhood mortality by two-third by 2015). Several studies have been conducted to identify the determinants of U5M. Studies have identified strong associations between maternal factors and childhood mortality. Maternal age has been shown to have a strong association with child survival [4-8]. However some of these studies did not consider certain important covariates such as sanitation, immunization, breastfeeding and previous child deaths in their research which might have confounding effect. Studies have also shown that children of educated mothers will survive better than children of non-educated mothers [5,9-15]. However another author has reported that maternal education has little or no effect on child survival [16].

Birth interval has been identified by numerous studies as a major determinant of childhood mortality [4,7,9,13,17-20] although not all of these studies have considered potential confounders like breastfeeding, intra-familial mortality risks (survival status of preceding child), and multiple pregnancy etc. Grand multiparity has been revealed to have a negative effect on child survival [10,15] Similarly, large family size has also been indicated to have a close association with poor rates of child survival. This was explained by increased intra-familiar competition for foods and other limited resources essential for child survival [13,19]. Availability of accessible health care services have been reported to have an inverse relationship with childhood mortality [4,10,21,22]. Despite these preponderance of evidence about the determinants of childhood mortality from different parts of the world, the majority of the studies did not consider the potential effects of some of the important confounders in their analyses [4-9,13,18,20]. Moreover, there are few good studies from Nigeria and most of which did not use national representative data [23-28]. Thus, the generalizability of the results is a serious problem in a country like Nigeria because of her diversity. The most recent national representative 2008 Nigerian Demographic and Health Survey (NDHS) [29] has estimated U5M rate at 157 children per 1,000 live births compared to their counterpart in the United Kingdom (England and Wales) which was said to be 2.8 children per 1,000 live births [30]. It means that 1 out of 6 children will before their fifth birthday in Nigeria [29]. It is therefore important to the explore the latest Nigeria Demographic and Health Survey data to identify the determinants responsible for the persistently high under-5 mortality in Nigeria. The objective of this study was to develop a predictive model and identify maternal, child, family and other risk factors associated U5M in Nigeria.

## Methods

### Setting

Nigeria is situated in the West Africa region with a total area of 923,768 kilometre square (km2), making it the fourteenth largest countries in Africa. Nigeria is lying between longitudes 2°40' and 14°41' E and latitudes 4°16' and 13°53' N. Nigeria shared boundary with Niger at the northern part, Benin Republic at the western part, Cameroon at the eastern part and the Atlantic Ocean at the southern part of the country. Nigeria is the most populous country in Africa and the most populous blacks' nation in the world. Nigeria population census 2006 was estimated at 140,431,790 with national growth rate of 3.2% per annum. The population density of Nigeria was estimated at 150 people per square kilometre. The country has over 250 ethnic groups [31,32] with varying languages and customs, creating a country of rich ethnic diversity. The ethnic groups are the Fulani/Hausa, Yoruba and Igbo accounting for 68% of the population while the Edo, Ijaw, Kanuri, Ibibio, Ebira, Nupe and Tiv comprise 27%. The remaining minorities accounted for the 5%. According to 2008 National Demographic and Health Survey, [29] children under -five accounted for 17.1% of the Nigeria population which makes every unit change in mortality to reflect great effect in the population. Also 33% of the total population is situated urban areas while 67% of the population were residing in rural areas. It has a GDP of about 200 billion United States dollars [33].

### Study design

This is a secondary analysis of population-based cross-sectional study which investigated the roles of child, maternal, paternal, family and other factors in U5M in Nigeria using the 2008 National Demographic and Health Survey data.

### Sampling technique

Detail information about the sampling technique that was applied for the data collection had been published in the final report of 2008 NDHS [29]. The NDHS was designed to yield a national representative data. Nigeria is made up of 36 states and Federal Capital Territory (FCT). Both states and FCT are subdivided into local government areas while each local government comprises of localities. The cluster unit was provided by the National Population Commission, based on the 2006 national census and is called a census enumeration area. Each locality comprises of census enumeration areas while each census enumeration area has at least 80 households (400 population). Stratified two-stage cluster randomised design was applied. The first stage was the allocation of 888 clusters among all the states and FCT. They were allocated among the states and FCT based on their size and rural/urban areas. The rural areas have 602 clusters while the urban areas got 286 clusters. Sample frame of households in each selected cluster was obtained and these households were randomly sampled from. At the end, the sampling procedure randomly selected a total of 36,800 households for an interview with a minimum of 950 and 41 households for interview in each state and cluster respectively. All women in each household within the age of 15-49 were interviewed; also all men age 15-59 in approximately half of the sampled households (16,800) were interviewed.

### Data collection

Detail information about the data collection and questionnaires has been published in the final report of 2008 NDHS [29]. Three types of questionnaires (household, women's and men's questionnaires) were used and face-to-face interviews were conducted. All women age 15-49 in each of the selected household were interviewed while all men within the age 15-59 in approximately half of the sampled households (16,800). Information was obtained about the children and their mothers and household. Retrospective information was taken about under-5 children that died in the last five years i.e. from 2003 to 2008. The exercise started in June 2008 and was completed in October 2008.

### Ethical consideration

Ethical approval for this project was obtained from Ethical Committee of ICF at Calverton, Maryland, USA and National Ethic Committee in Federal Ministry of Health, Abuja, Nigeria.

### Outcome variables

Each woman interviewed in the survey was asked to provide a detailed history of all her live births in chronological order. Under-five mortality was defined as the probability of dying before completing the fifth birthday.

### Explanatory variables

Selection of explanatory variables was based on previous studies [5,7,9,10,13,15,18,34-42] it is important to mention that analytical framework for child survival in developing country also gave good insight about the selection of the explanatory variables [43]. The following factors were included in the study: Maternal (current age, education, occupation, parity, marital status, age at first marriage, family planning, preceding birth interval, breastfeeding and health seeking behaviour) Childhood (sex, birth order, birth weight); Household (family size, sanitation number of wives, wealth index, fuel and water sources); Paternal factors (age, occupation); and other factors (place of residence, ethnicity and geopolitical region). The asset-based approach was used to asses wealth index. Also, we generated health seeking behaviour by combining 5 variables (having health care card, attended antenatal care, delivered in a health facility, knowledge of oral rehydration solution and received anti-tetanus) with the aid of Principal Component Analysis (PCA).

### Statistical analysis

Recoding and renaming were done for both Independent and dependent variables. One survival outcome variable was generated. Descriptive analysis of the variables was done. Univariable logistic regression was used to examine the association between the explanatory variables and the dependent outcome. Only explanatory variables that were statistically significant (P-value = 0.05) were incorporated into multivariable logistic regression. Likelihood Ratio test (LHR test) was used to test for the goodness of fit of the model. Receiver Operating Curve (ROC) was employed to examine the predictive power of the model. Hosmer-Lemeshow goodness-of-fit was used to examine the fitness of the model. Variance Inflation Factor (VIF) was used to check for multicollinearity. Predictive and complexity characteristics of the model were considered during modelling. Stata 11 software package was used for the analysis.

## Results

### Sample characteristics

Out of all the 28,647 children delivered by 28,647 mothers, 3,201 of these children died before their fifth birthday. This survey retrospectively covered from the year 2003 to 2008. Over 50% of the mothers interviewed have no former education and more than one quarter of them married before the age of 15 years. Use of contraception method is still a major problem in Nigeria, close to 90% of the mothers interviewed were not using any type of family planning methods.

Over 70% of the respondents were living in rural areas and approximately 90% of households were using coal, charcoal and wood for their cooking. The majority of the households have no toilet facility and only 10.5% have modern toilet facilities. More than 50% of people interviewed were poor and did not have access to safe water supply. Over 80% of the mothers interviewed have adopted birth interval of ≥ 18 months between their deliveries. Similarly, it was found that over 80% of the children were breastfed for more than 6 months. However approximately, one-third of the mothers were unemployed while almost half of the fathers interviewed were farmers and about two-third of them were having monogamous families. The result is shown Table 1

**Table 1:** Statistical summary of the explanatory variables

EXPLANATORY VARIABLES	SUMMARY	
	**NUMBER**	**PERCENTAGE (%)**

**Maternal factors**		

**Maternal education**		
No education	1,4418	50.3
Primary	6,552	22.9
Secondary or higher	7,677	26.8

**Maternal occupation**		
Not working	9,035	31.7
Business or cleric	9,985	35.1
Manual	9,470	33.2

**Parity**		
1	3,061	10.7
2, 3 and 4	13,759	48.0
≥ 5	11,827	41.3

**Marital status**		
Never married	506	1.8
Currently married	27,378	95.6
Formerly married	762	2.7

**Maternal age**		
≤ 20 years	3,319	11.6
21-25 years	6,787	23.7
26-30 years	8,157	28.5
31-35 years	5,043	17.6
> 35 years	5,341	18.6

**Maternal age at first marriage**		
< 15 years	7,967	28.3
15-19 years	12,706	45.2
20-24 years	5,243	18.6
≥ 25 years	2,225	7.9

**Family planning**		
No method	24,952	87.1
Folkloric/traditional	1,072	3.7
Modern	2,647	9.2

**Preceding birth interval**		
< 18 months	1,719	7.4
18-36 months	12,634	54.3
> 36 months	8,902	38.3

**Breastfeeding**		
< 6 months	4,722	17.5
6-12 months	6,266	23.2
> 12-18 months	8,629	32.0
> 18 months	7,351	27.3

**Health seeking behaviour**		
Very low	6,860	25.7
Low	8,862	23.3
Average	5,704	21.4
Good	5,224	19.6

**Child factors**		

**Sex**		
Male	14,604	51.0
Female	14,043	49.0

**Birth order**		
1,	5, 353	18.7
2, 3 or 4	13,069	45.6
≥ 5	10,225	35.7

**Birth weight**		
Large	13,012	46.5
Average	10,732	38.4
Small	4,239	15.1

**Household factors**		

**Family size**		
1-5	11,557	40.3
> 5	17,090	59.7

**Sanitation**		
Good toilet	2,955	10.5
Bad toilet	25,136	89.5

**Fuel sources**		
Gas	250	0.9
Kerosene	3,606	12.7
Other	24,518	86.4

**No of wives**		
One wife	18,063	66.4
Two or more wives	9,128	33.6

**Wealth index**		
Poor	14,475	50.5
Middle	5,609	19.6
Rich	8,563	29.9

**Water source**		
Safe water	13,021	46.3
Unsafe water	15,122	53.7

**Paternal age**		
< 22 years	264	1.0
22-30 years	5,453	20.3
> 30-40 years	10,854	40.4
> 40-50 years > 50 years	7,011	26.0
	3,277	12.2

**Paternal occupation**		
Business or cleric	7,815	28.1
Farming	12,645	45.5
Manual	7,312	26.3

**Others factors**		

**Residence**		
Rural	21,034	73.4
Urban	7,613	26.6

**Ethnicity**		
Major	16,838	59.1
Minor	11,643	40.9

**Region**		
North central	5,046	17.6
North east	6,559	22.9
North west	7,947	27.7
South east	2,450	8.6
South west	3,327	11.6
South south	3,318	11.6

### Univariable analyses

The results of univariable (unadjusted) analyses are shown in Table 2. Illiteracy, multi-parity and early pregnancy were inversely related to child survival. Odds of U5M among the children of mothers that have primary and secondary/higher education reduced by 17% and 42% respectively compared to children of mothers with no education. The likelihood of U5M among the children of mothers of parity of 2-4 and ≥ 5 increased by 60% and 131% respectively compared to children of nulliparous women; while odds of U5M among the children of mothers within the age of 21-25 years, 26-30 years and 31-35 years were reduced by 17%, 20% and 14% respectively compared to the children of mothers at the age of 20 years and below. Likelihood of U5M in children of mothers that were currently married and those that were formerly married increased by 38% and 104% compared to children of mothers that have never married.

**Table 2:** Result of univariable analysis logistic regression for under-5 mortality

EXPLANATORY VARIABLES			
	**OR**	**P-value**	**(C.I)**

**Maternal factors**			

**Maternal education**			
No education	1.00	-	(-)
Primary	0.83	0.001	(0.76-0.91)
Secondary or higher	0.58	0.001	(0.53-0.64)

**Maternal occupation**			
Not working	1.00	-	(-)
Business or cleric	0.96	0.380	(0.88-1.05)\
Manual	1.02	0.683	(0.93-1.12)

**Parity**			
1	1.00	-	(-)
2, 3 and 4	1.60	0.001	(1.37-1.87)
≥ 5	2.31	0.001	(1.98-2.70)

**Marital status**			
Never married	1.00	-	(-)
Currently married	1.38	0.047	(1.00-1.90)
Formerly married	2.04	0.001	(1.41-2.96)

**Maternal age**			
≤ 20 years	1.00	-	(-)
21-25 years	0.83	0.006	(0.73-0.95)
26-30 years	0.80	0.001	(0.71-0.91)
31-35 years	0.86	0.024	(0.75-0.97)
> 35 years	0.99	0.837	(0.87-1.24)

**Maternal age at first marriage**			
< 15 years	1.00	-	(-)
15-19 years	0.80	0.001	(0.74-0.87)
20-24 years	0.67	0.001	(0.60-0.75)
≥ 25 years	0.61	0.001	(0.52-0.72)

**Family planning**			
No method	1.00	-	(-)
Traditional	0.45	0.001	(0.35-0.58)
Modern	0.55	0.001	(0.47-0.65)

**Health Seeking Behaviour**			
Low	1.00	-	(-)
Average	1.17	0.001	(1.07-1.27)
High	0.55	0.001	(0.50-0.62)

**Preceding birth interval**			
< 18 months	1.00	-	(-)
18-36 months	0.49	0.001	(0.43-0.56)
> 36 months	0.30	0.001	(0.26-0.34)

**Breastfeeding**			
< 6 months	1.00	-	(-)
6-12 months	0.27	0.001	(0.24-0.30)
> 12-18 months	0.10	0.001	(0.08-0.11)
> 18 months	0.10	0.001	(0.08-0.11)

**Child factors**			

**Sex**			
Male	1.00	-	(-)
Female	0.87	0.001	(0.81-0.93)

**Birth order**			
1,	1.00	-	(-)
2, 3 or 4	0.87	0.011	(0.79-0.97)
≥ 5	1.25	0.001	(1.12-1.38)

**Birth weight**			
Average	1.00	-	(-)
Large	0.91	0.022	(0.83-0.99)
Small	1.39	0.001	(1.38-1.70)

**Paternal & Family factors**			

Family size			
1-5	1.00	-	(-)
> 5	0.77	0.001	(0.71-0.80)

**Sanitation**			
Good toilet	1.00	-	(-)
Bad toilet	1.98	0.001	(1.69-2.31)

**Fuel source**			
Gas	1.00	-	(-)
Kerosene	1.48	0.193	(0.82-2.69)
Others	2.66	0.001	(1.49-4.76)

**No of wives**			
One wive	1.00	-	(-)
More wives	1.35	0.001	(1.25-1.46)

**Wealth index**			
Poor	1.00	-	(-)
Rich	0.69	0.001	(0.64-0.74)

**Water source**			
Unsafe water	1.00	-	(-)
Safe water	0.76	0.001	(0.70-0.82)

**Paternal age**			
< 22 years	1.00	-	(-)
22-30 years	1.27	0.285	(0.82-1.96)
> 30-40 years	1.23	0.339	(0.80-1.90)
> 40-50 years	1.28	0.260	(0.83-1.98)
> 50 years	1.67	0.022	(1.08-2.59)

**Paternal occupation**			

Business/cleric	1.00	-	(-)
Farming	1.18	0.001	(1.08-1.29)
Manual	1.01	0.896	(0.91-1.12)

**Other factors**			

**Residence**			
Rural	1.00	-	(-)
Urban	1.53	0.001	(1.40-1.68)

**Ethnicity**			
Minors	1.00	-	(-)
Majors	1.02	0.546	(0.95-1.10)

**Regions**			
North central	1.00	-	(-)
North east	1.29	0.001	(1.15-1.45)
North west	1.37	0.001	(1.22-1.53)
South east	1.15	0.080 0.918	(0.98-1.34)
South west	0.99	0.001	(0.86-1.15)
South south	0.64		(0.54-0.75)

*** means variables omitted by StataIC 11 software package

Before controlling for other factors, maternal age at the first marriage was also found to have significant effect on U5M; the likelihood of U5M among the siblings of mothers that married at the age of 15-19 years, 20-24 years, and ≥ 25 years were found to reduce by 20%, 33% and 39% respectively compared to mothers that married before the age of 15 years. Similarly, odds of U5M among the children of mothers that embraced traditional and modern methods of contraception reduced by 55% and 45% respectively compared to mothers that did not use any contraception.

We found that health seeking behaviour has an impact on child survival; odds of having U5M among the siblings of mothers with the best health seeking behaviour reduced by 45% while those with average health seeking behaviour increased by 17% compared to the children of mothers with the worst health seeking behaviour. Adequate preceding birth interval, exclusive breastfeeding have protective effects on child survival. Likelihood of U5M among the siblings of mothers with a preceding birth interval of 18-36 months and > 36 months reduced by 51% and 70% respectively compared to mothers with a preceding birth interval of < 18 months. Being breastfed for 6-12 months, > 12-18 months and > 18 months reduced odds of U5M by 73%, 90%, and 90% respectively compared to children that were breastfed for < 6 months.

In the unadjusted analyses, being a female reduced odds of U5M by 13% while having birth order of 2-4 decreased odds of U5M by 13% but birth order of ≥ 5 increased its by 25% compared to children having birth order of one. Likelihood of U5M in children with low birth weight increased by 39% while that of large birth weight reduced by 9% compared to children that have normal birth weight.

Bad toilet facilities, cooking fuel and polygamous families were found to have positive association with U5M before considering other factors. Homes with bad toilet facilities increased odds of U5M by 98% compared to homes with good toilet facilities. Similarly, living in homes that were using charcoal as fuel source increased odds of U5M by 166% compared to those using gas. In similar manner, polygamous families increased odds of U5M by 35% compared to children from monogamous families.

Wealth, safe water and small family size were found to have protective effects on U5M before adjusting for all other factors. Likelihood of U5M in children from rich families reduced by 31% compared to children from poor family while safe water reduced odds of U5M by 24% compared to unsafe water. Families of 5 members or less decreased likelihood of U5M by 23% compared to families having > 5 members. Moreover, odds of U5M in rural areas increased by 53% compared to urban areas while odds of U5M were noticed to vary across the regions; in North East and North West it was observed to increase by 29% and 37% respectively but reduced by 36% in the South compared to North Central.

### Multivariable results

The results of multivariable analyses, where all factors were controlled for are shown in Table 3. Effect of maternal age at first marriage remained statistically significant. The likelihood of U5M among the siblings of women who married at age 20-24 years and ≥ 25 years declined by 20% and 30% respectively compared to women who married below age 15 years. Similarly, use of contraception, preceding birth interval and breastfeeding were found independently to have protective effects on U5M. The likelihood of U5M in children of mothers that were using a folkloric or traditional method of contraception reduced by 31% compared to children of mothers that did not embrace the use of contraception. Odds of U5M in children with 18-36 months and > 36 months of birth intervals reduced by 70% and 91% respectively compared to those delivered within birth interval < 18 months. These effects have trend. Breastfeeding found to reduce the odds of under-5 mortality by 57% in those that were breastfed for more > 18 months compared to those that were breastfed for < 6 months.

**Table 3:** Result of multivariable analysis logistic regression for under-5 mortality

EXPLANATORY VARIABLES			
	**OR**	**P-value**	**(C.I)**

**Maternal factors**			

**Maternal education**			
No education	1.00	-	(-)
Primary	0.98	0.742	(0.84-1.13)
Seconday or higher	1.13	0.232	(0.92-1.38)

**Parity**			
1	1.00	-	(-)
2, 3 and 4	***	***	(-)
≥ 5	***	***	(-)

**Marital status**	***	***	(-)

**Maternal age**			
≤ 20 years	1.00	-	(-)
21-25 years	1.23	0.113	(0.95-1.58)
26-30 years	1.70	0.001	(1.30-2.22)
31-35 years	2.48	0.001	(1.84-3.33)
> 35 years	2.87	0.001	(2.10-3.91)

**Maternal age at first marriage**			
< 15 years	1.00	-	(-)
15-19 years	***	***	(-)
20-24 years	0.80	0.001	(0.70-0.90)
≥ 25 years	0.70	0.001	(0.57-0.85)

**Family planning**			
No method	1.00	-	(-)
Traditional	0.69	0.017	(0.51-0.85)
Modern	***	***	(-)

**Health Seeking Behaviour**			
Low	1.00	-	(-)
Average	0.06	0.001	(0.05-0.07)
High	1.00	0.951	(0.88-1.12)

**Preceding birth interval**			
< 18 months	1.00	-	(-)
18-36 months	0.30	0.001	(0.26-0.34)
> 36 months	0.09	0.001	(0.07-0.10)

**Breastfeeding**			
< 6 months	1.00	-	(-)
6-12 months	1.00	0.955	(0.67-1.50)
> 12-18 months	0.90	0.373	(0.71-1.14)
> 18 months	0.43	0.001	(0.35-0.53)

**Child factors**			

**Sex**			
Male	1.00	-	(-)
Female	1.04	0.547	(0.92-1.17)

**Birth order**			
1,	1.00	-	(-)
2, 3 or 4	1.93	0.001	(1.56-2.37)
≥ 5	***	***	(-)

**Birth weight**			
Normal	1.00	-	(-)
Large	1.08	0.438	(0.87-1.27)
Small	1.31	0.004	(1.09-1.58)

**Household factors**			

Family size			
1-5	1.00	-	(-)
> 5	3.54	0.001	(3.07-4.08)

**Sanitation**			
Good toilet	1.00	-	(-)
Bad tiolet	1.77	0.001	(1.46-2.14)

**Fuel source**			
Gas	1.00	-	(-)
Kerosene	0.52	0.001	(0.44-0.63)
Others	0.28	0.001	(0.23-0.34)

**Wealth index**			
Poor	1.00	-	(-)
Rich	***	***	(-)

**Water source**			
Unsafe water	1.00	-	(-)
Safe water	***	***	(-)

**No of wives**			
One wife	1.00	-	(-)
More wives	1.47	0.001	(1.30-1.66)

**Paternal occupation**			
Business/cleric	1.00	-	(-)
Farming	0.82	0.121	(0.64-1.05)
Manual	0.87	0.328	(0.67-1.14)

**Other factors**			

**Residence**			
Urban	1.00	-	(-)
Rural	1.53	0.002	(1.16-2.00)

**Regions**			
North central	1.00	-	(-)
North east	0.75	0.514	(0.31-1.78)
North west	0.67	0.366	(0.28-1.60)
South east	0.36	0.001	(0.32-0.42)
South west	0.99	0.497	(0.67-1.22)
South south	0.96	0.583	(0.83-1.11)

*** means variables omitted by StataIC 11 software package

Birth order, toilet facility and birth weight of the children were independently associated with the U5M. Odds of U5M among children with low birth weight at birth increased by 31% compared to children with normal birth weight. Also the likelihood of U5M in homes with bad toilet facilities increased by 77% compared to homes with good toilet facilities. Similarly, odds of U5M in children with birth order of 2-4 increased by 93% compared to children with birth order of one. Despite the adjustment for confounders the independent effect of health seeking behaviour of the mother on child survival has shown that odds of U5M among the children of mothers with average health seeking behaviour reduced by 94% compared to children of mothers with worst health seeking behaviour.

Furthermore, in the multivariable model, large family size, residing in rural areas and having more than one wife, have negative impacts on child survival. It was found that residing in rural areas increased odds of U5M by 53% compared to children living in urban settlements. Similarly, Odds of U5M in children from families with more than 5 members increased by 254% compared to children from families with less than 5 members. In the same manner, having more than one wife has been shown to have positive associations with U5M. Odds of U5M increased by 47% in polygamous families compared to monogamous families. Child survival in different regions also varied. The likelihood of under-5 mortality in children living in South East, Nigeria reduced by 54% compared to children that were living in the North central part of Nigeria when other confounders have been considered. Contrary to expectation, the likelihood of U5M in children living in homes where kerosene and charcoal were sources of energy reduced by 48% and 72% respectively compared to those using gas.

### Model fit and Predictive power of the models

**Figure 1:**
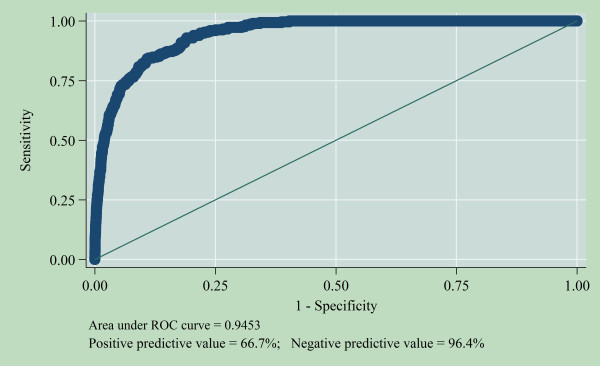
**Receiver operating curve (ROC) of multivariable model for predicting under-five mortality, Nigeria 2003-2008**.

Likelihood ratio test (LHR test) and Hosmer-Lemeshow goodness-of-fit were used to examine the fitness of the model. None of these raise any question concerning the fitness of the models. Variance inflation factor (VIF) was employed to check for multicollinearity. None of the VIF values were up to 10 and the mean VIF of the model was less than 6. It means there was no collinearity in the model (see additional file 1). Figure 1 shows the Receiver Operating Curve (ROC Curve). Area under the ROC curve was greater than 90% which means that the predictive power of the model was perfect. The positive predictive value was 66.7% and the negative predictive value was 96.4%.

## Discussion

The outcome of this study has shed more light on risk factors of U5M in Nigeria. This study has attempted to control for the effects of potential confounders by incorporating many factors into the analyses without over-modelling. Maternal age at first marriage was found to have an inverse relationship with U5M. This result shows that children of mothers that started childbearing at an early age were more prone to experience U5M more than those that commenced at an older age. This problem is now more pronounced because younger mothers formerly derived supports from mother in-laws and relatives that will stay with them for a long period of time but due to the economic crisis, this tradition has been eroded so young inexperience mothers have to take care of their children by themselves. Effect of maternal age at first marriage is consistent with other previous studies [18,44].

The likelihood of U5M reduced in children of mothers that embraced the use of contraception. This study revealed that U5M were less likely to occur in children of mothers that adopted folkloric or traditional method of contraception than those that were not using any contraception. Contraception has a way of militating against incidence of unwanted pregnancy and it enhances adequate child spacing. It ensures that only children that were planned for will come to life and these will improve their survival. This relationship did not contradict what prior studies have reported [8,13].

Birth interval was another important predictor of child survival. It has negative relationship with U5M. As child spacing increased, the likelihood of U5M reduced. This result supported what prior studies have reported [4,7]. Possible explanation for this is that mothers that waited for more than 18 months before having the next baby would have regained most body nutrients and blood loss during previous pregnancy and breast feeding. It is also a common medical knowledge that risk of obstetrics complications is higher in mothers that had short birth interval than those with long birth interval. Birth order was another determinant of child survival. It has a parallel association with U5M. The likelihood of U5M increased with increased in birth order of the child. This is consistent with other previous studies [5,45]. Possible mechanism for this is that as birth order increases, intra-familiar competition for foods and other limited resources essential for child survival will increase. Moreover, children are more prone to receive most impacts of it. Also as birth order increases level of child care reduces since the mother will have more children to care for.

It is important to mention that this study also revealed that children that were small at birth were more likely to die during before the age of 5 years than those that were having normal birth weight. A possible explanation for this, are the effects of preterm births. Most preterm children are more prone to have sepsis which is one of the leading causes of neonatal deaths. Other complications of preterm births are neonatal jaundice, apnoea etc. All these will account for high the likelihood of U5M in children with low birth weight. This is consistent with other previous studies [7,8,10]. This study has also shown that children raised in urban areas were more likely to survive better than those in rural areas. This finding supported what have been reported in other studies [4,9,46]. Likely explanation for this relationship is that children in urban areas have better access to health care services and all other essential health related services which are important for child survival.

This study revealed that good toilet facilities at home can reduce U5M and this is in line with other prior studies [21,47,48]. The outcome of this research has also shown that the number of wives that men married to, has a positive relationship with U5M. It was observed that as the number of wives increased, the likelihood of U5M in that family increased. This is consistent with previous study [49]. Possible reasons for this is intra-familiar competition for food and other resources which are essential for child survival. Family size was noticed to have an independent effect on U5M. This phenomenon can be explained, based on the fact that as family size increases, intra-familiar competition for foods and other essential services require for child survival increases, so also parental attention for their children decreases. As a result of these U5M will increase.

Health seeking of mothers has been shown to have a positive impact on child survival. It is common medical knowledge that children of mothers that were in a good habit of seeking medical attention will allow their children to have the privilege of receiving immunization and treatment of preventable childhood diseases. Studies have shown that these services reduced U5M [4,10]. However, the trend was not found in this relationship. It is essential to report that this research yielded two skewed results although this could be explained. It was noticed that U5M started to increase from in mothers in the age group 26-30 years and the odds of U5M increased as the age group increased. Prior studies have reported two contrary views about the effect of maternal age on U5M. Some research reported that maternal aged has an inverse relationship with U5M [23,26] while others revealed that it has no effect on U5M [7,50]. However, it is crucial to state that none of these studies considered maternal age at first marriage in their analyses, which was adjusted for in this study. Similarly, this study also revealed that child survival in homes that were using kerosene, fire woods and charcoals as sources of fuel would be better than those using gas. This was not in agreement with previous study [51]. Possible explanation for this is that, < 1% of the studied population were using gas.

Moreover survival of children living in South west of Nigeria was better than those residing in North central of Nigeria. Children in North central of Nigeria were more likely to die before their 5th year birthday than their counterparts in North east of Nigeria. Prior study has also reported regional variations in child survival [52]. Possible explanation for these regional differences in child survival can be attributed to variations in vegetation across the regions. Children tend to survive better in regions with better agricultural output than regions with low output. Also the political will of government to implement good health policies varies across the regions.

### Strengths and weaknesses

It is important to mention the strength and weaknesses of this study. This study is a population based cross sectional study with a large sample size that was randomly selected in order to reflect the true studied population. Thus, the findings of this study can be generalized to the studied population and to any other country's population similar to Nigeria. Possibility of selection and sampling bias in this study have been minimized because the whole country's population was considered before implementing stratified two-stage random sampling technique. Also the percentage of missing data in this study were negligible. However is also important to stress the weaknesses in this type of study. In this type of study, causal effect cannot be measured. Also the analysis of this study was based on point prevalence data; it is not possible to know whether the data are time dependent or not although the predictive power of the model have clarified this area. Generally, length bias is a common problem with this type of study.

### Recommendation

U5M in Nigeria is an urgent public health issue and appropriate measures are needed to be taken. This study has provided insights into the risk factors of U5M in Nigeria and this will be vital information for health policy makers in government and non-governmental organisation. Nigerian government and non-governmental organisation need to adopt programmes that will empower women because maternal factors were part of the major determinants of childhood mortality in Nigeria. Implementation of health programmes that will encourage breastfeeding, use of contraception and child spacing will go a long way in curtailing U5M in Nigeria.

Reorientation of parents concerning their health seeking behaviour via implementation of good health promotion programmes is highly essential to reduce U5M. It is important to mention that the Federal Ministry of Health, Nigeria needs to work in collaboration with other health related ministries such as the Federal Ministry of Agriculture, Education, Labour, Water and Federal Ministry of Women Affairs in order to reduce childhood mortality. This is because the provision of jobs and empowerment of women will have positive impacts on child survival. Enactment of laws that will ensure good sanitation should be in place in order to improve child survival.

Health inequality is another area that government and non-governmental organisation need to give urgent attention. This will improve equity among different regions and between rural and urban communities in Nigeria. In addition case-control study can be used to further investigate the effects of some of the factors. Maintaining small family size and encouraging monogamous family should be encouraged and promoted.

## Conclusions

This study has revealed that maternal, child and family and other factors were the important determinants of U5M in Nigeria. It will be of great benefit if all these predictors could be properly considered during planning, formulation and implementation of policies that will improve child survival.

## Competing interests

The authors declare that they have no competing interests.

## Authors' contributions

GAK was involved in the conception of the study. GAK carried out data extraction. GAK conducted statistical analysis with contributions from VTA and OAU. GAK drafted the paper with contributions from VTA and OAU and GAK. All authors read and approved the final manuscript.

## Pre-publication history

The pre-publication history for this paper can be accessed here:

http://www.biomedcentral.com/1471-2393/12/10/prepub

## Supplementary Material

Additional file 1 **Result of test for multicollinearity in the multivariable model for under-5 mortality**. Click here for file
