# Supplementary material for: Risk factors and a predictive model for under-five mortality in Nigeria: evidence from Nigeria demographic and health survey
Source: BMC Pregnancy Childbirth. 2012 Feb 29;12:10. doi: 10.1186/1471-2393-12-10 (PMC3313900; doi:10.1186/1471-2393-12-10)
Supplement: Additional file 1 — Result of test for multicollinearity in the multivariable model for under-5 mortality. [file 1471-2393-12-10-S1.PDF]

TABLE 4: RESULT OF MULTIVARIATE ANALYSIS LOGISTIC REGRESSION

EXPLANATO-RY VARIABLES	UNDER-5		
	OR	P-value	(C.I)
**Maternal factors**			
**Maternal education**			
No education	1.00	-	(-)
Primary	0.98	0.742	(0.84 – 1.13)
Secondary or higher	1.13	0.232	(0.92 – 1.38)
**Parity**			
1	1.00	-	(-)
2, 3 and 4	***	***	(-)
≥5	***	***	(-)
**Marital status**	***	***	(-)
**Maternal age**			
≤ 20 years	1.00	-	(-)
21 – 25 years	1.23	0.113	(0.95 – 1.58)
26 – 30 years	1.70	0.001	(1.30 – 2.22)
31 – 35 years	2.48	0.001	(1.84 – 3.33)
> 35 years	2.87	0.001	(2.10 – 3.91)
**Maternal age at first marriage**			
< 15 years	1.00	-	(-)
15 – 19 years	***	***	(-)
20 – 24 years	0.80	0.001	(0.70 – 0.90)
≥ 25 years	0.70	0.001	(0.57 – 0.85)
**Family planning**			
No method	1.00	-	(-)
Traditional	0.69	0.017	(0.51 – 0.85)
Modern	***	***	(-)
**Health Seeking Behaviour**			
Low	1.00	-	(-)
Average	0.06	0.001	(0.05 – 0.07)
High	1.00	0.951	(0.88 – 1.12)
**Preceding birth interval**			
< 18 months	1.00	-	(-)
18 – 36 months	0.30	0.001	(0.26 – 0.34)
> 36 months	0.09	0.001	(0.07 – 0.10)
**Breastfeeding**			
< 6 months	1.00	-	(-)
6 – 12 months	1.00	0.955	(0.67 – 1.50)
>12 – 18 months	0.90	0.373	(0.71 – 1.14)
>18 months	0.43	0.001	(0.35 – 0.53)
**Child factors**			
**Sex**			
Male	1.00	-	(-)
Female	1.04	0.547	(0.92 – 1.17)
**Birth order**			
1	1.00	-	(-)

EXPLANATO-RY VARIABLES	UNDER-5		
	OR	P-value	(C.I)
2, 3 or 4	1.93	0.001	(1.56 – 2.37)
≥5	***	***	(-)
**Birth weight**			
Normal	1.00	-	(-)
Large	1.08	0.438	(0.87 – 1.27)
Small	1.31	0.004	(1.09 – 1.58)
**Paternal & family factors**			
**Family size**			
1 – 5	1.00	-	(-)
>5	3.54	0.001	(3.07 – 4.08)
**Sanitation**			
Good toilet	1.00	-	(-)
Bad tiolet	1.77	0.001	(1.46 – 2.14)
**Fuel source**			
Gas	1.00	-	(-)
Kerosene	0.52	0.001	(0.44 – 0.63)
Others	0.28	0.001	(0.23 – 0.34)
**Wealth index**			
Poor	1.00	-	(-)
Rich	***	***	(-)
**Water source**			
Unsafe water	1.00	-	(-)
Safe water	***	***	(-)
**No of wives**			
One wife	1.00	-	(-)
More wives	1.47	0.001	(1.30 – 1.66)
**Paternal occupation**			
Business / cleric	1.00	-	(-)
Farming	0.82	0.121	(0.64 – 1.05)
Manual	0.87	0.328	(0.67 – 1.14)
**Other factors**			
**Residence**			
Urban	1.00	-	(-)
Rural	1.53	0.002	(1.16 – 2.00)
**Regions**			
North central	1.00	-	(-)
North east<br>North west	0.75<br>0.67	0.514<br>0.366	(0.31 – 1.78)<br>(0.28 – 1.60)
South east	0.36	0.001	(0.32 – 0.42)
South west	0.99	0.497	(0.67 – 1.22)
South south	0.96	0.583	(0.83 – 1.11)

*** means variables omitted by StataIC 11 software package
